# Free combination of dutasteride plus tamsulosin for the treatment of benign prostatic hyperplasia in South Korea: analysis of drug utilization and adverse events using the National Health Insurance Review and Assessment Service database

**DOI:** 10.1186/s12894-021-00941-1

**Published:** 2021-12-21

**Authors:** Zrinka Lulic, Hwancheol Son, Sang-Bae Yoo, Marianne Cunnington, Pratiksha Kapse, Diane Miller, Vanessa Cortes, Suna Park, Rachel H. Bhak, Mei Sheng Duh

**Affiliations:** 1 GlaxoSmithKline, Brentford, Middlesex UK; 2 Department of Urology, Seoul National University College of Medicine, Boramae Hospital, Seoul, Korea; 3 GlaxoSmithKline, Seoul, Korea; 4 GlaxoSmithKline, Mumbai, India; 5 GlaxoSmithKline, Collegeville, PA USA; 6 GlaxoSmithKline, Bogotá, Colombia; 7 Analysis Group, Inc., Boston, MA USA

## Abstract

**Objective:**

To assess the use and safety of free combination therapy (dutasteride and tamsulosin), dutasteride monotherapy, or tamsulosin monotherapy in patients with benign prostatic hyperplasia (BPH).

**Methods:**

This non-interventional retrospective cohort study used claims data from the Korea Health Insurance Review and Assessment-National Patient Sample database. Patients with BPH ≥ 40 years of age receiving combination therapy (dutasteride 0.5 mg and tamsulosin 0.4 mg daily) or dutasteride 0.5 mg, or tamsulosin 0.4 mg daily dose between 2012 and 2017 were included. The frequency, duration of treatment and risk of any adverse event (AE) or serious AE (SAE) was compared for combination therapy versus each monotherapy using non-inferiority testing.

**Results:**

Of 14,755 eligible patients, 1529 (10.4%) received combination therapy, 6660 (45.1%) dutasteride monotherapy, and 6566 (44.5%) tamsulosin monotherapy. The proportion of patients treated with combination therapy exceeded the pre-specified 3% threshold for ‘frequent’ use. Safety results indicated a similar risk of any AE and SAE irrespective of treatment group. The adjusted relative risk for any AE over the treatment observation period comparing combination therapy with dutasteride monotherapy was 1.07 (95% confidence interval [CI] 1.03, 1.12), and with tamsulosin monotherapy was 0.98 (95% CI 0.95, 1.02) demonstrating non-inferiority. The adjusted relative risk for any SAE was 1.07 (95% CI 0.66, 1.74) and 0.90 (95% CI 0.56, 1.45), compared with dutasteride and tamsulosin monotherapy, respectively. Although the SAE results did not statistically demonstrate non-inferiority of combination therapy based on pre-specified margins, the 95% CI for the risk ratio estimates included the null with a lower limit below the non-inferiority margins, indicating no meaningful differences in SAE risk between groups. Absolute SAE risks were low.

**Conclusion:**

Combination therapy with dutasteride and tamsulosin is frequently used in real-world practice in South Korea for treatment of BPH and demonstrates a safety profile similar to either monotherapy.

**Supplementary Information:**

The online version contains supplementary material available at 10.1186/s12894-021-00941-1.

## Introduction

Benign prostatic hyperplasia (BPH) is one of the most common non-malignant conditions in older men [1, 2]. A nationwide survey from the United States (US) reported BPH prevalence of 25% in men > 50 years of age [3]. In a population-based, cross-sectional survey conducted in the US, the United Kingdom (UK) and Sweden, symptoms suggestive of possible BPH were highly prevalent in men, reported in up to 46% of the population studied [4]. Notably, BPH prevalence increases with age, from 14.8% in men aged 40–49 years to 36.8% in those ≥ 80 years [1]. Although current data are limited for Southeast Asia, the overall incidence of BPH in South Korea was reported to be 2105 per 100,000 men based on data from patients diagnosed with BPH in 2008, using a nationwide South Korean database, Health Insurance Review and Assessment (HIRA) [5]. As expected, the prevalence of BPH increased with age; the highest incidence was in patients ≥ 70 years of age.

A common manifestation of BPH is lower urinary tract symptoms (LUTS), including difficulty in voiding, and nocturia [6, 7]. These have considerable negative impacts on health-related quality of life and sexual functioning [6, 8, 9]. The short-term aim of LUTS/BPH therapy is to provide relief of symptoms by improving flow of urine [10]; long-term treatment goals are to alleviate bothersome LUTS, prevent acute urinary retention, and reduce the risk of complications [7, 11, 12].

A fixed-dose combination of dutasteride 0.5 mg plus tamsulosin 0.4 mg therapy (5α-reductase inhibitor (5-ARI) and α1-blocker) is currently approved in over 90 countries, including the US, UK, and Australia, for symptomatic BPH [13–15]. Fixed-dose combination therapy has only recently been approved in South Korea (May 2021) [16], although for several years now the Korean Urological Association guidelines for BPH have endorsed α1-blocker plus 5-ARI combination therapy as being more effective than α1-blocker monotherapy for improving LUTS [17]. The availability of real-world data representative of national populations offers the opportunity to generate country-specific evidence on the benefit:risk of new medicine indications and combinations more rapidly than was previously possible through more traditional clinical study approaches.

The current study assessed real-world use and safety of dutasteride 0.5 mg and tamsulosin 0.4 mg in free combination (ie, administered concomitantly) therapy among patients with BPH using the South Korean HIRA claims database.

## Methods

### Study design

**Fig. 1:**
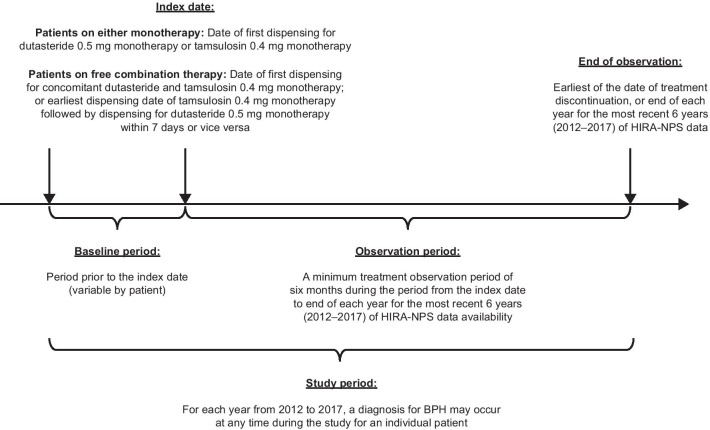
Study design. BPH, benign prostatic hyperplasia; HIRA-NPS, Health Insurance Review and Assessment Service-National Patient Sample

This was a non-interventional retrospective cohort study using claims data from the HIRA-National Patient Sample (HIRA-NPS) database. The NPS database comprises random 3% annual samples from the overall HIRA database with patients followed for a maximum of 1 year. Eligible patients from each year (2012 − 2017) were pooled into one population. More details are provided in Additional file 1. From the overall population, three treatment cohorts were defined: patients treated with free combination therapy (dutasteride 0.5 mg and tamsulosin 0.4 mg daily), dutasteride 0.5 mg monotherapy (0.5 mg once daily), and tamsulosin 0.4 mg monotherapy (0.4 mg once daily or 0.2 mg twice daily). The study period comprised the baseline period, index date (administration of therapy), and observation period (Fig. 1).

This non-interventional retrospective study was exempted from Institutional Review Board or ethics review committee approval. Patients were not contacted, and patient data were anonymized in compliance with current privacy laws and data de-identification guidelines in South Korea. The study sponsor did not have access to patient identifiers. Additional study information is available in the GlaxoSmithKline Clinical Study Register, Study ID Number: 212907.

### Study population

Patients were included from the HIRA-NPS database from 2012 to 2017 if they had: at least one medical claim with a primary or secondary diagnosis for BPH at any time during the year; ≥ 1 prescription dispensed for free combination therapy, dutasteride 0.5 mg monotherapy or tamsulosin 0.4 mg monotherapy; were ≥ 40 years of age on the index date; and were observed for ≥ 6 months while receiving treatment. Patients were excluded from the analysis if they had a record of a primary diagnosis of BPH-related surgery (no specific surgical procedure codes were available), or if they had a medical claim with a primary or secondary diagnosis for prostate cancer, during the baseline period.

### Study objectives

The primary objectives were to describe the frequency and duration of treatment, overall risk of any adverse event/serious adverse event (AE/SAE) during the treatment observation period, and the demographic and clinical characteristics of prevalent patients with BPH at treatment initiation.

Evaluated AEs were restricted to those that were identifiable with Korean Classification of Disease codes and were included in the global datasheet for dutasteride-tamsulosin hydrochloride (Additional file 2). SAEs were any condition from the list of AEs that was the primary diagnosis associated with hospitalization or death during the observation period.

Prostate cancer was included in the global datasheet under ‘warnings’ as an area of potential risk associated with 5α-reductase inhibitors that needs to be monitored (ie, not a confirmed drug-related AE).

### Statistical analysis

Inverse-probability-of-treatment weighting (IPTW) was used to adjust for imbalances in the distribution of baseline characteristics across all cohorts before conducting comparisons. Standardized differences in baseline characteristics of < 10% were deemed as denoting meaningful balance across treatment cohorts [18].

Frequency and duration of treatment with free combination therapy and each monotherapy were summarized using descriptive statistics. In a previous study, the prevalence of moderate or severe LUTS among patients with BPH was reported to be 17.4%, and 33.7% of those patients received treatment for BPH [19]. Based on this it was estimated that 5.9% of patients would be eligible for receiving a BPH medical treatment in the population of interest. In the absence of a pre-defined threshold, this estimate was halved (ie, 3%) and used as a threshold to define frequent use for the free combination therapy of dutasteride and tamsulosin in our study population.

To assess safety, non-inferiority testing with a one-sided α-error level of 0.025 was conducted to test the hypothesis that the overall risk of any AE or SAE with free combination therapy was no greater than with either monotherapy. Risk ratios (RRs) for any AE and any SAE were estimated using IPTW-adjusted log-binomial regression models, with a single variable for the treatment cohort. Robust variance estimators were used to obtain the corresponding 95% confidence intervals (CIs). Non-inferiority was confirmed if the upper limit of the 95% CIs of the RRs fell below the pre-specified non-inferiority margin of 1.64 and 1.30 for AEs and 0.84 and 0.77 for SAEs for comparisons with dutasteride and tamsulosin monotherapy, respectively. Non-inferiority margins were based on results from the subgroup of South Korean patients in the pivotal CombAT trial. The RR values for any AE for patients receiving free combination therapy compared with dutasteride 0.5 mg monotherapy and with tamsulosin 0.4 mg monotherapy were 2.7 and 1.7, respectively. In addition, the RR values for any SAE for patients receiving free combination therapy compared with dutasteride 0.5 mg monotherapy and tamsulosin 0.4 mg monotherapy were 0.7 and 0.6, respectively. The non-inferiority margins were estimated using the point-estimate method and taking a preserved effect of 50% of the log RRs, which is in line with regulatory guidance [20, 21].

A subgroup analysis by age (40–59, 60–69, and ≥ 70 years of age), sensitivity analysis among patients without AEs or SAEs during the baseline period, and evaluation of specific AEs using superiority testing with a two-sided α-level of 0.05 were also conducted. No adjustments were made for multiple comparisons. Analyses were conducted using SAS Enterprise Guide Software Version 7.1 (SAS Institute Inc., Cary, NC).

## Results

### Patient population

A total of 246 720 patients with ≥ 1 medical claim for a primary diagnosis for BPH were identified from the HIRA-NPS database from January 1, 2012 to December 31, 2017. Of these, 14,755 (6.0%) patients were eligible and included in the analysis (Additional file 3): 1529 (10.4%) patients received daily treatment with free combination therapy, 6660 (45.1%) received dutasteride monotherapy 0.5 mg daily, and 6566 (44.5%) received tamsulosin monotherapy 0.4 mg daily.

Prior to IPTW adjustment, several baseline demographic and clinical characteristic imbalances were observed between treatment groups with standardized differences of 10% or above. Patients treated with free combination therapy were more likely to have polyuria (13.9% vs 9.9%, standardized difference = 12.6%), a higher number of symptoms or findings associated with BPH (0.3% vs 0.2%, standardized difference = 11.0%), and BPH with LUTS (17.9% vs 12.3%, standardized difference = 15.7%), than those treated with dutasteride 0.5 mg. The free combination and tamsulosin groups were more balanced; however, patients treated with free combination therapy were less likely to be 50–59 years of age (7.7% vs 12.9%, standardized difference = 17.3%) and more likely to be ≥ 70 years of age (60.6% vs 51.6%, standardized difference = 18.3%) at baseline, than those treated with tamsulosin 0.4 mg.

Following IPTW adjustment, baseline demographics and clinical characteristics were balanced across all treatment cohorts with standardized differences of < 10% (Table 1). Most patients (> 87%) were aged ≥ 60 years; BPH with LUTS affected 13.3% to 18.6% of patients, and polyuria was the most common symptom associated with BPH, affecting 10.6% to 14.6% of the IPTW-adjusted samples. The most common medications used during the baseline period were non-steroidal anti-inflammatory drugs (NSAIDS; 34.6−37.9%), calcium channel blockers (21.0−24.2%), and antihypertensives (21.8–23.4%).

**Table 1:** Baseline characteristics of patients treated with free combination therapy compared with those treated with dutasteride 0.5 mg or tamsulosin 0.4 mg monotherapy, after adjustment using inverse probability of treatment weight

	Free combination therapy versus dutasteride monotherapy			Free combination therapy versus tamsulosin monotherapy		
	Free combination of dutasteride plus tamsulosin therapy (n = 1527)	Dutasteride monotherapy (n = 6661)	Std. diff* (%)	Free combination of dutasteride plus tamsulosin therapy (n = 1544)	Tamsulosin monotherapy (n = 6574)	Std. diff* (%)
Age, n (%)						
40–49 years	29 (1.9)	150 (2.2)	2.3	20 (1.3)	90 (1.4)	0.7
50–59 years	165 (10.8)	704 (10.6)	0.8	182 (11.8)	777 (11.8)	0.2
60–69 years	488 (32.0)	2133 (32.0)	0.1	517 (33.5)	2201 (33.5)	0.0
≥ 70 years	844 (55.3)	3675 (55.2)	0.3	826 (53.5)	3506 (53.3)	0.3
Clinical characteristics, n (%)						
Any AE^†^	422 (27.7)	1857 (27.9)	0.5	476 (30.8)	2030 (30.9)	0.1
Cardiovascular disease^‡^	565 (37.0)	2475 (37.2)	0.3	622 (40.3)	2612 (39.7)	1.2
Hyperlipidemia	304 (19.9)	1361 (20.4)	1.3	338 (21.9)	1433 (21.8)	0.3
Chronic pulmonary disease	221 (14.5)	988 (14.8)	0.9	258 (16.7)	1088 (16.5)	0.4
BPH with LUTS	210 (13.7)	889 (13.3)	1.1	288 (18.6)	1193 (18.2)	1.2
Polyuria^§^	167 (10.9)	709 (10.6)	1.0	225 (14.6)	936 (14.2)	1.0
Concomitant medications, n (%)						
NSAIDs	530 (34.7)	2302 (34.6)	0.4	585 (37.9)	2417 (36.8)	2.3
Calcium channel blockers	330 (21.6)	1400 (21.0)	1.4	374 (24.2)	1500 (22.8)	3.3
Antihypertensives	334 (21.9)	1454 (21.8)	0.1	361 (23.4)	1459 (22.2)	2.8

AE, adverse event; BPH, benign prostatic hyperplasia; LUTS, lower urinary tract symptoms; NSAIDs, nonsteroidal anti-inflammatory drugs;

Std. diff, standardized difference

^*^For continuous variables, the standardized difference was calculated by dividing the absolute difference in means of the free combination therapy cohort and reference monotherapy cohorts by the pooled standard deviation (SD) of both groups, for each comparison. The pooled SD was the square root of the average of the squared SD. For dichotomous variables, the standardized difference was calculated using the following equation where P is the respective proportion of participants in each treatment cohort: [(P_freecombination therapy_- P_reference_)/ √(P_freecombinationtherapy_x(1 – P_freecombinationtherapy_) + P_reference_ x (1 – P_reference_))/ 2]

^†^For the purpose of this analysis, the data on prostate cancer were included in any AE. See Additional file 2 for list of AEs

^‡^Three categories of Quan–Charlson comorbidities (ie, congestive heart failure, peripheral vascular disease, and myocardial infarction) are listed under cardiovascular disease

^§^Polyuria includes nocturia and urinary frequency

### Treatment frequency and duration

The proportion of patients treated with free combination therapy was 10.4%, compared with 45.1% (dutasteride monotherapy) and 44.5% (tamsulosin monotherapy); this exceeded the threshold of 3% selected to define frequent combined use. The mean ± standard deviation (SD) duration of treatment was similar for patients treated with free combination therapy (292.5 ± 54.1 days) and either monotherapy (297.1 ± 54.0 days for dutasteride; 295.8 ± 54.0 days for tamsulosin) (Table 2).

**Table 2:** Frequency and duration of treatment with free combination therapy, dutasteride 0.5 mg monotherapy, or tamsulosin 0.4 mg monotherapy in patients with prevalent BPH in South Korea

		Free combination therapy versus dutasteride monotherapy		Free combination therapy versus tamsulosin monotherapy	
	Free combination of dutasteride plus tamsulosin therapy (n = 1529)	Dutasteride monotherapy (n = 6660)	Std. diff^*^	Tamsulosin monotherapy (n = 6566)	Std. diff^*^
Treatment duration (days)					
Mean ± SD	292.5 ± 54.1	297.1 ± 54.0	8.6	295.8 ± 54.0	6.1
Median, IQR	305.0 (249.0, 341.0)	310.0 (260.0, 342.0)		310.0 (255.0, 343.0)	
Treatment duration, n (%)					
6–9 months	519 (33.9)	2020 (30.3)	7.7	2085 (31.8)	4.7
9–12 months	1010 (66.1)	4640 (69.7)	7.7	4481 (68.2)	4.7

BPH, benign prostatic hyperplasia; IQR, interquartile range; SD, standard deviation; Std. Diff, standardized difference

^*^For continuous variables, the standardized difference was calculated by dividing the absolute difference in means of the free combination therapy cohort and reference monotherapy cohorts by the pooled standard deviation (SD) of both groups, for each comparison. The pooled SD was the square root of the average of the squared SD. For dichotomous variables, the standardized difference was calculated using the following equation where P is the respective proportion of participants in each treatment cohort: [(P_freecombination therapy_- P_reference_)/ √(P_freecombinationtherapy_x(1 – P_freecombinationtherapy_) + P_reference_ x (1 – P_reference_))/ 2]

### Overall risk of any AE or SAE

**Fig. 2:**
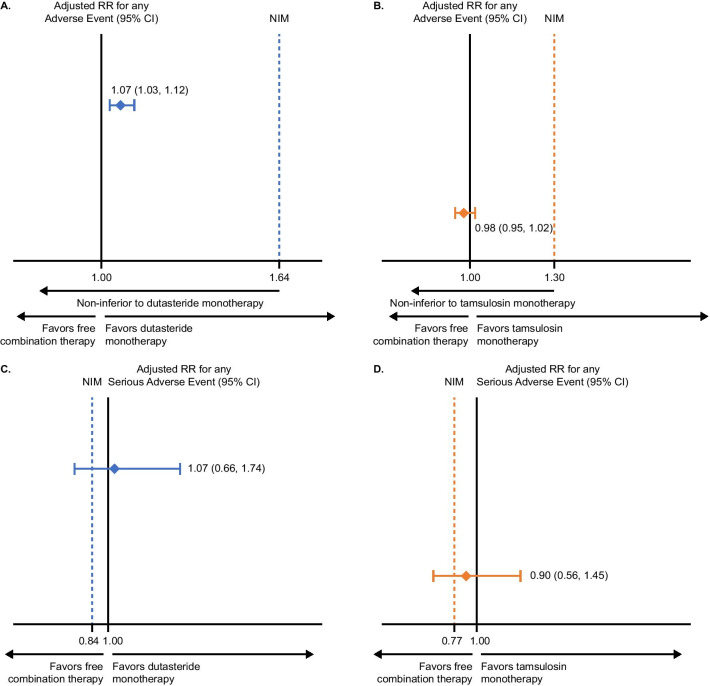
Risk of any AE or SAE among patients with prevalent BPH receiving free combination therapy compared with dutasteride 0.5 mg monotherapy (**A**: AE, **C**: SAE) or tamsulosin 0.4 mg monotherapy (**B**: AE, **D**: SAE). For the purpose of this analysis, the data on prostate cancer were included in the risk calculations for any AE and any SAE. AE, adverse event; BPH, benign prostatic hyperplasia; CI, confidence interval; NIM, non-inferiority margin; RR, risk ratio; SAE, serious adverse event

The risk of any AE occurring was 71.5% (free combination therapy), 64.6% (dutasteride 0.5 mg) and 71.6% (tamsulosin 0.4 mg). The adjusted RR for any AE over the treatment observation period comparing free combination therapy with dutasteride 0.5 mg monotherapy was 1.07 (95% CI 1.03, 1.12) (Fig. 2A) and comparing free combination therapy with tamsulosin 0.4 mg monotherapy was 0.98 (95% CI 0.95, 1.02) (Fig. 2B). The risk of any AE with free combination therapy was non-inferior to that of both monotherapy groups based on non-inferiority margins (non-inferiority *P* < 0.001 each comparison).

The incidence of any SAE in the overall patient population was rare and similar across treatment groups: 1.6% (free combination therapy), 1.3% (dutasteride 0.5 mg monotherapy), and 1.7% (tamsulosin 0.4 mg monotherapy). The adjusted RR for any SAE over the treatment observation period comparing free combination therapy with dutasteride 0.5 mg monotherapy was 1.07 (95% CI 0.66, 1.74) (Fig. 2C), and comparing free combination therapy with tamsulosin 0.4 mg monotherapy was 0.90 (95% CI 0.56, 1.45) (Fig. 2D). Non-inferiority was not shown for either comparison (non-inferiority *P* = 0.852 and 0.762 for comparison with dutasteride and tamsulosin monotherapies, respectively). However, the 95% CI for the RR estimate included the null value of one and had a lower limit below the non-inferiority margins, indicating no meaningful differences in the risk of SAE between treatment groups.

### Sensitivity analysis among patients without baseline AEs or SAEs

Results relating to the frequency and duration of treatment and risk of any AE or SAE were consistent with the primary results when patients with AEs or SAEs during the baseline period were excluded from the analysis. For comparison of AEs with free combination therapy versus dutasteride 0.5 mg monotherapy, the adjusted RR was 1.10 (95% CI 1.04, 1.16), indicating a small meaningful difference in favor of dutasteride monotherapy. For comparison of free combination therapy with tamsulosin 0.4 mg monotherapy, the adjusted RR for any AE was 0.98 (95% CI 0.93, 1.04), indicating no meaningful difference. The RR for any SAE of free combination therapy versus dutasteride 0.5 mg monotherapy was 1.13 (95% CI 0.58, 2.24), and versus tamsulosin 0.4 mg monotherapy was 0.80 (95% CI 0.42, 1.51), indicating no meaningful differences.

### Subgroup analysis by age

Treatment frequency with free combination therapy was lowest in patients 40–59 years of age (6.3%) and highest in patients > 70 years of age (11.7%). Among patients aged 60–69 years, 9.8% used free combination therapy. The average duration of treatment was similar for patients treated with free combination therapy and each monotherapy across all age groups (Additional file 4). The risk of any AE and SAE within each age category was similar to that for the overall population, with no meaningful differences between treatment groups (Additional file 5).

### Overall risk of specific AEs and SAEs

The most common AEs throughout the treatment observation period among free combination therapy, dutasteride 0.5 mg monotherapy, and tamsulosin 0.4 mg monotherapy were constipation (26.2%, 19.1%, 24.3%, respectively), depressed mood (14.3%, 11.9%, 15.6%, respectively), urticaria (14.1%, 12.4%, 15.6%, respectively) and dizziness (13.2%, 12.5%, 13.5%, respectively) (Table 3). The 95% CI of the RR for most specific AEs overlapped with 1, indicating no meaningful differences between treatment groups.

**Table 3:** Risk of specific AEs with free combination therapy compared with dutasteride or tamsulosin monotherapy

		Free combination therapy versus dutasteride monotherapy		Free combination therapy versus tamsulosin monotherapy	
	Free combination of dutasteride plus tamsulosin therapy (n = 1529)	Dutasteride monotherapy (n = 6600)	Adjusted RR (95% CI)*	Tamsulosin monotherapy (n = 6566)	Adjusted RR (95% CI)*
Specific AE, n (%)					
Constipation	401 (26.2)	1271 (19.1)	1.31 (1.17, 1.45)	1595 (24.3)	1.05 (0.94, 1.16)
Depressed mood	219 (14.3)	794 (11.9)	1.05 (0.90, 1.22)	1025 (15.6)	0.91 (0.78, 1.05)
Urticaria	216 (14.1)	828 (12.4)	1.02 (0.87, 1.18)	1024 (15.6)	0.89 (0.76,1.03)
Dizziness	202 (13.2)	833 (12.5)	1.01 (0.87, 1.19)	889 (13.5)	0.98 (0.84, 1.14)
Arrhythmia	188 (12.3)	608 (9.1)	1.33 (1.13, 1.57)	638 (9.7)	1.24 (1.05, 1.46)
Vertigo	176 (11.5)	643 (9.7)	1.20 (1.01, 1.42)	735 (11.2)	1.05 (0.89, 1.24)
Diarrhea	166 (10.9)	702 (10.5)	0.99 (0.83, 1.17)	765 (11.7)	0.94 (0.80, 1.12)
Pruritus	146 (9.5)	624 (9.4)	0.98 (0.81, 1.18)	757 (11.5)	0.83 (0.69, 1.00)
Cardiac failure	137 (9.0)	400 (6.0)	1.37 (1.12, 1.68)	442 (6.7)	1.23 (1.01, 1.49)
Vomiting	117 (7.7)	374 (5.6)	1.29 (1.03, 1.62)	513 (7.8)	0.99 (0.80, 1.22)
Rhinitis	103 (6.7)	422 (6.3)	1.11 (0.89, 1.39)	454 (6.9)	0.97 (0.78, 1.22)
Dyspnea	74 (4.8)	266 (4.0)	1.10 (0.84, 1.44)	288 (4.4)	1.00 (0.77, 1.30)
Asthenia	51 (3.3)	196 (2.9)	1.06 (0.77, 1.47)	204 (3.1)	1.06 (0.75, 1.50)
Localized edema	30 (2.0)	130 (2.0)	0.84 (0.55, 1.29)	149 (2.3)	0.80 (0.52, 1.21)
Impotence	21 (1.4)	88 (1.3)	1.15 (0.69, 1.93)	108 (1.6)	1.09 (0.66, 1.79)
Epistaxis	19 (1.2)	84 (1.3)	1.10 (0.65, 1.87)	87 (1.3)	1.08 (0.60, 1.95)
Syncope orthostatic	13 (0.9)	62 (0.9)	0.83 (0.45, 1.53)	70 (1.1)	0.74 (0.41, 1.34)
Hypotension	11 (0.7)	35 (0.5)	1.34 (0.67, 2.70)	34 (0.5)	1.24 (0.62, 2.46)
Rash	9 (0.6)	22 (0.3)	1.45 (0.65, 3.26)	23 (0.4)	1.33 (0.57, 3.09)
Alopecia	5 (0.3)	34 (0.5)	0.87 (0.33, 2.28)	28 (0.4)	1.09 (0.41, 2.91)
Breast disorder	5 (0.3)	25 (0.4)	1.12 (0.39, 3.19)	18 (0.3)	1.53 (0.47, 4.99)
Dry mouth	5 (0.3)	17 (0.3)	0.98 (0.34, 2.83)	23 (0.4)	0.83 (0.30, 2.31)
Visual impairment	2 (0.1)	16 (0.2)	0.44 (0.10, 1.97)	17 (0.3)	0.41 (0.10, 1.79)
Other specified disorders of male genital organ	1 (0.1)	8 (0.1)	0.92 (0.12, 7.37)	10 (0.2)	0.54 (0.07, 4.25)
Vision blurred	0 (0.0)	5 (0.1)	–	7 (0.1)	–
Angioedema	0 (0.0)	4 (0.1)	–	2 (0.0)	–
Erythema multiforme	0 (0.0)	2 (0.0)	–	4 (0.1)	–
Premature ejaculation	0 (0.0)	2 (0.0)	–	1 (0.0)	–
Breast cancer	0 (0.0)	1 (0.0)	–	1 (0.0)	–
Hypertrichosis	0 (0.0)	1 (0.0)	–	0 (0.0)	–
Loss of libido	0 (0.0)	1 (0.0)	–	0 (0.0)	–
Dermatitis exfoliative	0 (0.0)	0 (0.0)	–	3 (0.0)	–
Priapism	0 (0.0)	0 (0.0)	–	1 (0.0)	–

AE, adverse event; CI, confidence interval; RR, risk ratio.*RRs of any or specific AEs among patients receiving free combination therapy compared to each monotherapy were estimated using log-binomial regression models adjusted for inverse probability of treatment weights. A robust variance estimator was used to derive the 95% CIs

The risk of specific SAEs was low (< 1% each) in all treatment groups. The most commonly reported SAEs in the free combination therapy, dutasteride monotherapy and tamsulosin monotherapy groups, respectively, were dizziness (0.5%, 0.3%, 0.4%), cardiac failure (0.3%, 0.1%, 0.2%), arrhythmia (0.2%, 0.2%, 0.2%), and vertigo (0.1%, 0.2%, 0.2%).

### Prostate cancer

Prostate cancer was observed in 12.6%, 9.4%, and 12.0% of patients in the free combination therapy, dutasteride 0.5 mg monotherapy, and tamsulosin 0.4 mg monotherapy groups, respectively, throughout the treatment observation period.

## Discussion

This retrospective, real-world study was based on a large sample of patients from the South Korean HIRA-NPS database, which includes information on 50 million patients and covers 98% of the total population through the universal coverage system [22]. Although previous studies have assessed BPH treatments using Korean national databases [23–25], to our knowledge this is the first study to analyze the use and safety of free combination therapy (dutasteride and tamsulosin), dutasteride monotherapy, and tamsulosin monotherapy within the Korean population. It also shows the potential for real-world data to inform benefit:risk assessments outside of traditional clinical trial approaches that may capture relatively small country-specific population numbers. Dutasteride and tamsulosin are widely administrated as a free combination therapy outside of Korea [13–15]. Similarly, the results from our analyses demonstrated frequent use of this free combination therapy for treatment of BPH in the Korean population, without increased risk of any AE or SAE in comparison to either monotherapy. These findings enhance our understanding of BPH treatment in a country with an aging population and elevating prescription for BPH medication [24, 26]. Additionally, these findings support the current evidence from clinical trials on the safety of free combination therapy for treatment of BPH as well as underscoring the benefits of this treatment.

Our analysis demonstrates use of free combination dutasteride 0.5 mg and tamsulosin 0.4 mg was in 10.4% of patients with BPH, which is classified as frequent use (> 3%), based on pre-specified criteria. This is consistent with a previous analysis in South Korea using the HIRA database from 2007–2011, which found that 12–17% of newly diagnosed patients with BPH were prescribed combination therapy [25]. Importantly, combination therapy with an α1-blocker and a 5-ARI is recommended by international BPH treatment guidelines [27, 28] as well as the 2016 Korean clinical practice guideline for BPH [17]. In Korea, tamsulosin 0.4 mg has only recently been approved for use in patients with BPH and there has since been an increase in the use of combination therapy that includes this dose [29, 30]. Continued increase in the use of the combination of dutasteride 0.5 mg and tamsulosin 0.4 mg is expected, particularly given the low satisfactory relief of symptoms observed in patients using tamsulosin 0.2 mg [31]. However, studies have shown inferior medication compliance with free- versus fixed-dose combination therapy [32, 33]. Therefore, clinical outcomes may improve for patients with BPH by increasing compliance and lessening the pill burden on an aging population through fixed-dose combination, which has recently been approved in Korea.

In this analysis, the risk of any AE was similar for those treated with free combination therapy and dutasteride or tamsulosin monotherapies as demonstrated through non-inferiority testing and various subgroup and sensitivity analyses. The risk of any SAE was uniformly low. These results are consistent with the CombAT study, which observed a similar safety profile between combination and monotherapy of dutasteride and tamsulosin [34]. The most common AEs in the CombAT study were related to sexual function; however, these waned over time. Although our study did not assess AEs longitudinally, there was a numerically higher adjusted relative risk of impotence and breast disorders in the combination groups; however, no statistical analysis was applied to these data. Nonetheless, the risk of any AE and SAE was similar between combination and monotherapy, which extends the evidence of the acceptability of the safety profiles for the dutasteride 0.5 mg and tamsulosin 0.4 mg combination in the BPH population from a real-world setting in South Korea.

Our study has some limitations. Although IPTW-adjustment was used for comparisons of safety endpoints, the potential for bias may remain. For example, the HIRA-NPS database does not include information on variables that influence the choice of BPH treatment strategy, such as prostate volume, total serum prostate-specific antigen, and LUTS. This may lead to unmeasured or residual confounding of data interpretation. Furthermore, key information may have been missed as the baseline period for capturing covariates differed across patients and was short. Additionally, this study relied on diagnosis codes associated with medical claims to determine a BPH diagnosis, clinical characteristics, AEs and SAEs. As such, any miscoding within the database may have resulted in misclassification of BPH clinical characteristics, AEs (particularly of milder, self-limiting events), and SAEs. Furthermore, the HIRA database does not include information on whether AEs and SAEs were treatment related, which may have resulted in miscoding or AEs and SAEs not being reported in this study. This was mitigated by evaluating specific AEs and SAEs related to the therapies under investigation in this study, based on prior clinical trials, post-marketing surveillance studies, and real-world studies [14]. Despite these potential limitations that are associated with real-world data, the results were comparable to those from randomized controlled trials (for example, CombAT and CONDUCT) that would not be subject to the same biases.

The strengths of this study are the generalizability of the findings to the wider BPH population in South Korea resulting from the large sample size and use of the most commonly used database in South Korea, the robustness of the comparative analyses through IPTW, inclusion of subgroup analyses, and consistency with available BPH literature [22].

The results of this study based on real-world evidence suggest that dutasteride 0.5 mg and tamsulosin 0.4 mg free combination is frequently used in South Korea for the treatment of BPH and the safety profile for free combination therapy is similar to either monotherapy.

## Supplementary Information

**Additional file 1**. Health Insurance Review and Assessment-National Patient Sample (HIRA-NPS) claims database.

**Additional file 2**. Adverse events from the Global Datasheet for dutasteride-tamsulosin hydrochloride.

**Additional file 3**. Identification of treatment cohorts for eligible patients with BPH from the South Korean HIRA-NPS database.

**Additional file 4**. Frequency and duration of treatment with free combination therapy, dutasteride monotherapy, or tamsulosin monotherapy among patients with prevalent BPH in South Korea by age group.

**Additional file 5**. Risk of AEs and SAEs with free combination therapy compared with dutasteride or tamsulosin monotherapy*, by patient age group.

## Data Availability

GSK makes available anonymized individual participant data and associated documents from interventional clinical studies, which evaluate medicines upon approval of proposals submitted to http://www.clinicalstudydatarequest.com. To access original data for studies that have been re-analyzed, other types of GSK sponsored research, for study documents without patient-level data and for clinical studies not listed, please submit an enquiry via the website.

## Abbreviations

5-ARI: 5α-reductase inhibitor
AE: adverse event
BPH: benign prostatic hyperplasia
CI: confidence interval
HIRA: Health Insurance Review and Assessment
IPTW: inverse-probability-of-treatment weighting
IQR: interquartile range
LUTS: lower urinary tract symptoms
NPS: National Patient Sample
RR: risk ratio
SAE: serious adverse event
SD: standard deviation
UK: United Kingdom
US: United States
