# Supplementary material for: Free combination of dutasteride plus tamsulosin for the treatment of benign prostatic hyperplasia in South Korea: analysis of drug utilization and adverse events using the National Health Insurance Review and Assessment Service database
Source: BMC Urol. 2021 Dec 21;21:178. doi: 10.1186/s12894-021-00941-1 (PMC8691067; doi:10.1186/s12894-021-00941-1)
Supplement: Supplementary file 1 — Additional file 1. Health Insurance Review and Assessment-National Patient Sample (HIRA-NPS) claims database. [file 12894_2021_941_MOESM1_ESM.docx]

**Additional file 1:** Health Insurance Review and Assessment-National Patient Sample (HIRA-NPS) claims database

The HIRA claims database accounts for over 98% of the total population claims in South Korea from almost 80,000 healthcare service providers across the country. The HIRA-NPS database was chosen for this analysis, as it represents a stratified random annual sample of approximately 3% of the total HIRA patient population of over a million patients and includes comprehensive information for each patient including diagnosis, treatment, procedures, surgical history, and prescription drugs. Additionally, as the HIRA-NPS database randomly samples only about 3% of patients from HIRA each year, it was assumed that patients selected each year were independent and there was no overlap between each year of HIRA-NPS data used in this study. Furthermore, patients are followed for a maximum of 1 year in the HIRA-NPS database and therefore, patients from each of the 6 years of data availability (2012–2017) represent distinct patient groups.
