# Supplementary material for: Free combination of dutasteride plus tamsulosin for the treatment of benign prostatic hyperplasia in South Korea: analysis of drug utilization and adverse events using the National Health Insurance Review and Assessment Service database
Source: BMC Urol. 2021 Dec 21;21:178. doi: 10.1186/s12894-021-00941-1 (PMC8691067; doi:10.1186/s12894-021-00941-1)
Supplement: Supplementary file 2 — Additional file 2. Adverse events from the Global Datasheet for dutasteride-tamsulosin hydrochloride. [file 12894_2021_941_MOESM2_ESM.docx]

**Additional file 2:** Adverse events from the Global Datasheet for dutasteride-tamsulosin hydrochloride

The list of adverse events (AEs) was determined from the AEs and warnings sections identified in the Global Datasheet for dutasteride-tamsulosin hydrochloride [[14](#_ENREF_14)], based on results of the CombAT trial, other clinical trials, observational studies, and post-marketing studies. In addition, to facilitate their identification in the HIRA-NPS database, the list of AEs was restricted to conditions that were identifiable with Korean Classification of Disease codes.

Alopecia

Angioedema

Arrhythmia

Asthenia

Breast cancer

Breast disorder

Cardiac failure

Constipation

Depressed mood

Dermatitis exfoliative

Diarrhea

Dizziness

Dry mouth

Dyspnea

Epistaxis

Erythema multiforme

Hypertrichosis

Impotence

Localized edema

Loss of libido

Orthostatic hypotension

Other specified disorders of male genital organ

Premature ejaculation

Priapism

Prostate cancer

Pruritus

Rash

Rhinitis

Syncope

Urticaria

Vertigo

Vision blurred

Visual impairment

Vomiting
