# Supplementary material for: Free combination of dutasteride plus tamsulosin for the treatment of benign prostatic hyperplasia in South Korea: analysis of drug utilization and adverse events using the National Health Insurance Review and Assessment Service database
Source: BMC Urol. 2021 Dec 21;21:178. doi: 10.1186/s12894-021-00941-1 (PMC8691067; doi:10.1186/s12894-021-00941-1)
Supplement: Supplementary file 3 — Additional file 3. Identification of treatment cohorts for eligible patients with BPH from the South Korean HIRA-NPS database. [file 12894_2021_941_MOESM3_ESM.docx]

## Additional file 3: Identification of treatment cohorts for eligible patients with BPH from the South Korean HIRA-NPS database.

| **Selection criteria** | | n | % of previous step |
| --- | --- | --- | --- |
| Step 1 | Patients with at least one medical claim with a primary or secondary diagnosis for BPH at any time during the year | 246,720 | _ |
| Step 2 | At least one dispensing for free combination therapy, dutasteride 0.5 mg monotherapy, or tamsulosin 0.4 mg monotherapy | 38,643 | 15.7 |
| Step 3 | At least 40 years of age at the index date | 38,293 | 99.1 |
| Step 4 | A minimum treatment observation period of 6 months | 14,955 | 39.1 |
| Step 5 | No record of a BPH-related surgery during the baseline period, as identified by a primary diagnosis for BPH | 14,931 | 99.8 |
| Step 6 | No medical claim with a primary or secondary diagnosis for prostate cancer during the baseline period | 14,755 | 98.8 |
| **Eligible patients (N=14,755)** | | **n** | **%** |
| Free combination therapy | | 1529 | 10.4 |
| Dutasteride monotherapy | | 6660 | 45.1 |
| Tamsulosin monotherapy | | 6566 | 44.5 |

BPH, benign prostatic hyperplasia; HIRA-NPS, Health Insurance Review and Assessment Service, National Patient Sample.
