# Supplementary material for: Free combination of dutasteride plus tamsulosin for the treatment of benign prostatic hyperplasia in South Korea: analysis of drug utilization and adverse events using the National Health Insurance Review and Assessment Service database
Source: BMC Urol. 2021 Dec 21;21:178. doi: 10.1186/s12894-021-00941-1 (PMC8691067; doi:10.1186/s12894-021-00941-1)
Supplement: Supplementary file 4 — Additional file 4. Frequency and duration of treatment with free combination therapy, dutasteride monotherapy, or tamsulosin monotherapy among patients with prevalent BPH in South Korea by age group. [file 12894_2021_941_MOESM4_ESM.docx]

## Additional file 4: Frequency and duration of treatment with free combination therapy, dutasteride monotherapy, or tamsulosin

monotherapy among patients with prevalent BPH in South Korea by age group.

|  |  | **Free combination therapy vs dutasteride monotherapy** | | **Free combination therapy vs tamsulosin monotherapy** | |
| --- | --- | --- | --- | --- | --- |
| **Age category** | **Free combination of dutasteride plus tamsulosin therapy**  **(N=1529)** | **Dutasteride monotherapy**  **(N=6660)** | **Std. diff*** | **Tamsulosin monotherapy**  **(N=6 566)** | **Std. diff*** |
| **Age 40–59 years** |  |  |  |  |  |
| Number of patients on treatment, n | 126 | 920 | _ | 947 | _ |
| Treatment duration (days) |  |  |  |  |  |
| Mean ± SD | 284.2 ± 55.7 | 286.1 ± 55.5 | 3.3 | 291.5 ± 54.7 | 13.2 |
| Median, IQR | 291.5 (236.0, 338.0) | 297.5 (237.0, 337.0) |  | 305.0 (248.0, 341.0) |  |
| Duration of treatment, n (%) |  |  |  |  |  |
| 6–9 months | 56 (44.4) | 363 (39.5) | 10.1 | 334 (35.3) | 18.8 |
| 9–12 months | 70 (55.6) | 557 (60.5) | 10.1 | 613 (64.7) | 18.8 |
| **Age 60–69 years** |  |  |  |  |  |
| Number of patients on treatment, n | 476 | 2161 | _ | 2233 | _ |
| Treatment duration (days) |  |  |  |  |  |
| Mean ± SD | 288.3 ± 54.6 | 295.3 ± 53.8 | 13 | 295.3 ± 54.3 | 13 |
| Median, IQR | 300.5 (242.0, 337.0) | 310.0 (254.0, 342.0) |  | 310.0 (254.0, 342.0) |  |
| Duration of treatment, n (%) |  |  |  |  |  |
| 6–9 months | 173 (36.3) | 700 (32.4) | 8.3 | 710 (31.8) | 9.6 |
| 9–12 months | 303 (63.7) | 1461 (67.6) | 8.3 | 1523 (68.2) | 9.6 |
| **Age ≥70 years** |  |  |  |  |  |
| Number of patients on treatment, n | 927 | 3579 | _ | 3386 | _ |
| Treatment duration (days) |  |  |  |  |  |
| Mean ± SD | 295.9 ± 53.4 | 301.0 ± 50.2 | 9.9 | 297.4 ± 53.5 | 2.8 |
| Median, IQR | 309.0 (255.0, 344.0) | 314.0 (270.0, 342.0) |  | 311.0 (258.0, 344.0) |  |
| Duration of treatment, n (%) |  |  |  |  |  |
| 6–9 months | 290 (31.3) | 957 (26.7) | 10.0 | 10 41 (30.7) | 1.2 |
| 9–12 months | 637 (68.7) | 2 622 (73.3) | 10.0 | 2 345 (69.3) | 1.2 |

BPH, benign prostatic hyperplasia; IQR, interquartile range; SD, standard deviation; Std. diff, standardized difference.
*For continuous variables, the standardized difference was calculated by dividing the absolute difference in means of the free combination therapy cohort and reference monotherapy cohorts by the pooled SD of both groups, for each comparison. The pooled SD was the square root of the average of the squared SD. For dichotomous variables, the standardized difference was calculated using the following equation where P is the respective proportion of participants in each treatment cohort: [(P_freecombination therapy_- P_reference_)/ √(P_freecombinationtherapy_x(1 – P_freecombinationtherapy_) + P_reference_ x (1 – P_reference_))/ 2.
