# Supplementary material for: Free combination of dutasteride plus tamsulosin for the treatment of benign prostatic hyperplasia in South Korea: analysis of drug utilization and adverse events using the National Health Insurance Review and Assessment Service database
Source: BMC Urol. 2021 Dec 21;21:178. doi: 10.1186/s12894-021-00941-1 (PMC8691067; doi:10.1186/s12894-021-00941-1)
Supplement: Supplementary file 5 — Additional file 5. Risk of AEs and SAEs with free combination therapy compared with dutasteride or tamsulosin monotherapy*, by patient age group. [file 12894_2021_941_MOESM5_ESM.docx]

## Additional file 5: Risk of AEs and SAEs with free combination therapy compared with dutasteride or tamsulosin monotherapy*, by patient age group.

| **Age category** |  | **Free combination therapy vs  dutasteride monotherapy** | | **Free combination therapy vs  tamsulosin monotherapy** | |
| --- | --- | --- | --- | --- | --- |
|  | **Free combination of dutasteride plus tamsulosin therapy,**  **n/N (%)** | **Dutasteride**  **monotherapy, n/N (%)** | **Adjusted RR**  **(95% CI)**^†^ | **Tamsulosin monotherapy, n/N (%)** | **Adjusted RR**  **(95% CI)**^†^ |
| **Age 40–59 years** | | | | | |
| Any AE | 75/126 (59.5) | 419/920 (45.5) | 1.21 (0.98, 1.50) | 565/947 (59.7) | 1.10 (0.90, 1.34) |
| Any SAE^‡^ | 2/126 (1.6) | 5/920 (0.5) | 2.19 (0.33, 14.43) | 6/947 (0.6) | 1.88 (0.28, 12.61) |
| **Age 60–69 years** | | | | | |
| Any AE | 299/476 (62.8) | 1309/2161 (60.6) | 1.02 (0.94, 1.12) | 1554/2233 (69.6) | 0.91 (0.84, 0.98) |
| Any SAE^‡^ | 4/476 (0.8) | 18/2161 (0.8) | 1.22 (0.38, 3.97) | 30/2233 (1.3) | 0.68 (0.24, 1.93) |
| **Age ≥70 years** | | | | | |
| Any AE | 719/927 (77.6) | 2575/3579 (71.9) | 1.07 (1.02, 1.12) | 2584/3386 (76.3) | 1.02 (0.98, 1.06) |
| Any SAE^‡^ | 19/927 (2.0) | 66/3579 (1.8) | 0.88 (0.52, 1.50) | 76/3386 (2.2) | 0.81 (0.48, 1.36) |

AE, adverse event; BPH, benign prostatic hyperplasia; CI, confidence interval; RR, risk ratio; SAE, serious adverse event.

*RRs of any AEs or SAEs among BPH patients receiving free combination therapy compared to each monotherapy were estimated using log-binomial regression models adjusted for inverse probability of treatment weights.

^†^A robust variance estimator was used to derive the 95% CIs.

^‡^SAEs were defined as the primary diagnosis that was associated with a hospitalization or death during the observation period.
